# Synthesis and Characterization of Novel Ternary-Hybrid Nanoparticles as Thermal Additives

**DOI:** 10.3390/ma16010173

**Published:** 2022-12-24

**Authors:** Jalal Mohammed Zayan, Abdul Khaliq Rasheed, Akbar John, Waleed Fekry Faris, Abdul Aabid, Muneer Baig, Batoul Alallam

**Affiliations:** 1 Department of Mechanical Engineering, International Islamic University Malaysia, Kuala Lumpur 50728, Selangor, Malaysia; 2 Department of New Energy Science and Engineering, School of Energy and Chemical Engineering, Xiamen University Malaysia, Jalan Sunsuria, Bandar Sunsuria, Sepang 43900, Selangor, Malaysia; 3 Environmental Technology Division, School of Industrial Technology, Universiti Sains Malaysia, Gelugor 11800, Penang, Malaysia; 4 Renewable Biomass Transformation Cluster, School of Industrial Technology, Universiti Sains Malaysia, Gelugor 11800, Penang, Malaysia; 5 Department of Engineering Management, College of Engineering, Prince Sultan University, P.O. Box 66833, Riyadh 11586, Saudi Arabia; 6 Advanced Medical and Dental Institute, Universiti Sains Malaysia, Bertam, Kepala Batas 13200, Penang, Malaysia

**Keywords:** nanofluids, heat transfer fluid, thermal conductivity, ternary hybrid

## Abstract

The performance of water as a heat transfer medium in numerous applications is limited by its effective thermal conductivity. To improve the thermal conductivity of water, herein, we report the development and thermophysical characterization of novel metal-metal-oxide-carbon-based ternary-hybrid nanoparticles (THNp) GO-TiO_2_-Ag and rGO-TiO_2_-Ag. The results indicate that the graphene oxide- and reduced graphene oxide-based ternary-hybrid nanoparticles dispersed in water enhance the base fluid (H_2_O) thermal conductivity by 66% and 83%, respectively, even at very low concentrations. Mechanisms contributing to this significant enhancement are discussed. The experimental thermal conductivity is plotted against the existing empirical hybrid thermal conductivity correlations. We found that those correlations are not suitable for the metal-metal-oxide-carbon combinations, calling for new thermal conductivity models. Furthermore, the rheological measurements of the nanofluids display non-Newtonian behavior, and the viscosity reduces with the increase in temperature. Such behavior is possibly due to the non-uniform shapes of the ternary-hybrid nanoparticles.

## 1. Introduction

Removal of excess heat from engines, power-plant equipment, manufacturing processes, electronics, and transmission systems has been challenging. Water, ethylene glycol, and mineral oils are commonly used heat transfer fluids known as base fluids. These base fluids have lower thermal conductivity, thus limiting their performance and applications. Thermal conductivity in base fluids can be enhanced by adding or dispersing solid particles such as metallic, non-metallic, or carbon, a concept first demonstrated by Maxwell in 1881 [1]. He dispersed micro-sized particles in liquid to study the thermal conductivity enhancement, which gave him limited success. It was noticed that the dispersed micro-sized particles settled down after some time due to their higher density. Recent scientific advancements have opened the opportunity to produce nano-sized particles suspended for a longer time in the fluids due to the low density. The nanomaterials are interestingly smaller and different compared to the materials of other sizes at a larger scale. Hence, nanoparticles’ characteristics, shape, size, design characteristics, and production in controlled physical structures are quite challenging, but are advantageous considering that they have a larger surface area compared to materials of a significantly larger size with the same mass and volume [2]. This advantage makes the nanoparticles more reactive to chemical and thermal changes. This nanoscale advantage opens a huge opportunity to unlock its advantageous potential for application in various fields. When dispersed into any base fluids, this higher surface area of the nanoparticles makes the solid–liquid interaction more effective. Nanofluids usually have higher thermal conductivity as desired for efficient thermal energy transfer in most systems. Studies have shown that the coefficient of thermal conductivity increases considerably in the nanofluids, even with a very low concentration of nanoparticles compared to the standard base fluids [3]. Therefore, nanofluidics could replace the existing coolants in several sectors, such as energy, electronics, transportation, and manufacturing [4].

The nanofluids’ heat transfer enhancement is greatly influenced by factors such as the concentration of the nanoparticles, particle sizes, particle materials, particle shapes, and types of base fluids. It is always desirable to have a higher stability of nanoparticles in the standard base fluid to achieve a lasting thermal conductivity enhancement. A few nanofluids show a more significant enhancement in the thermal conductivity performance despite containing a minimal concentration of nanoparticles in it (ϕ < 1%) [5]. The thermal conductivity enhancements differ based on the type of nanoparticles used in nanofluids. Several studies were performed on single-material nanoparticle-based nanofluids in the past few decades, and the results are well-established. Modern-day applications require a combination of high thermal conductivity, stable rheological properties, a longer stability time, and homogeneity. These characteristics can be achieved by hybridizing the single-material nanoparticles with other nanoparticles [6].

### The Rationale behind the Ternary-Hybrid Synthesis

Although different hybrids have emerged since the past decade, new nanoparticle combinations continue to evolve due to advancements in the synthesis routes and application needs. The composition of the binary-hybrid and ternary-hybrid nanoparticles (THNp) is selected according to the desired outcome based on the application requirements. For example, ZnO-Ag/water nanofluids were studied and found to increase base fluids’ thermal conductivity at a concentration of 2% at 50 °C [7]. TiO_2_-MWCNTs in water-ethylene glycol hybrid nanofluid enhanced the thermal conductivity by 38.7% at 0.05 °C to 1% at 20 to 50 °C [8]. TiO_2_-SiO_2_ at a concentration of 1% in various combinations shows enhancement in the thermal conductivity of base fluids [9]. Likewise, countless studies are reporting the enhancement of thermal conductivity of the base fluid with the addition of hybrid nanoparticles in various combinations [10]. The hybrid nanoparticle combination aimed for in this study is silver, titanium dioxide, and graphene nanoparticles, which are metal, metal oxide, and carbon materials. The rationale behind this combination is to offer maximum thermal conductivity enhancement, a neutral chemical effect, and increased yield in a molecular tool called polymerase chain reaction (PCR). PCR is a vital tool for molecular diagnostics, involving amplifying a target DNA through a series of rapid heating–cooling cycles.

The synthesis of the novel ternary-hybrid (tri-hybrid) nanoparticles (THNp) consists of three well-known nanoparticles. The selection of the different nanoparticles was based on their thermophysical and rheological properties. Then, the individual nanoparticles are decorated on each other to form ternary-hybrid nanoparticles (THNp). As a result, the THNp has many advantages, such as high thermal conductivity, better stability in base fluids, a large surface area, crystallinity, and zeta potential. However, inadequate studies to investigate carbon-based ternary-hybrid nanoparticles have been performed until now. 

Graphene is an atom-thick sheet of sp^2^ carbon atoms arranged in a hexagonal pattern attached to the carbon atoms present in a honeycomb crystal mesh structure. The graphene oxide and the reduced graphene oxide are functional groups of graphene. In the present study, the graphene oxide-based nanoparticles are combined with two other metallic nanoparticles, titanium dioxide and silver. Both silver and titanium dioxide nanoparticles are well-established in the literature [11]. Graphene is a unique material famous for its exceptional properties, particularly when it comes to its high thermal conductivity and larger surface area. Graphene is hydrophobic, which does not dissolve easily in water and other water-based solvents. It can be made hydrophilic by attaching suitable functional groups to form graphene oxide [12]. Graphene oxide (GO) is then heavily oxygenated with other functional groups that contain oxygen, such as epoxide hydroxyl, carbonyl, and carboxyl groups. The interfacial interaction of the polar polymer matrices is higher due to the functional groups. Reduced graphene oxide (rGO) nevertheless has a lesser amount of other functional groups that contain oxygen. Chemical and thermal reduction methods can be employed to produce reduced graphene oxide. Hydrazine hydrate and ascorbic acid are usually the preferred reducing agents used in the preparation of the rGO. Different reduction agents will afford different levels of the carbon to oxygen ratio and chemical composition. The second nanoparticle used in the preparation of the THNp is silver. The linear and nonlinear optical properties of silver nanoparticles are controlled by many factors, such as size, shape, and inter-particle spacing, and environment, spectral, and geometrical properties. Silver nanoparticles are used in many fields, including detecting heavy metals in water and as coatings for glass slides. In developing analytical sensors, silver nanoparticles are used as excellent conductors of heat. They are used as thermal conductivity enhancers in various fluid media such as ethylene glycol, water, and oils. They are mainly used because of their stability without forming sedimentation for more prolonged periods [13].

Titanium dioxide (TiO_2_) is another nanoparticle used in the preparation of the THNp. TiO_2_ is one of the transitional metal oxides and one of the most widely used nanoparticles in numerous applications. Many methods can be used to synthesize titanium dioxide, which is vastly used as a thermal conductivity enhancer in various applications such as refrigerants, pool boiling, conduction enhancers, convective heat transfer, and antifogging coatings’ light-reflective properties. Another advantage is its adaptability to high-pressure applications, with varying concentrations as a desired property [14]. 

The novelty of the current study relates to the selection, synthesis, and characterization of two ternary-hybrid nanoparticles containing a distinct combination of metal-metal oxide-carbon nanoparticles and their nanofluids by dispersing in water as a base fluid in very low concentrations (ϕ < 1%). This type of ternary-hybrid combination in nanoparticles has not been studied to date. The novel ternary-hybrid nanoparticle aggregates containing graphene oxide-titanium oxide-silver nanoparticles decoration are used to enhance the effective thermal conductivity of the base fluids. The characterization of the THNPs was performed using scanning electron microscopy (SEM), Fourier-transform infrared spectroscopy (FTIR), Raman spectroscopy, X-ray diffraction (XRD), and zeta potential. The colloids were then studied for their thermal conductivity characteristics and rheological properties. Finally, the experimental measurements were compared with numerous hybrid theoretical correlation models. 

In summary, this study aims to form ternary hybrids of graphene and metal oxides; secondly, to establish thermo-physical properties of the formulated nanofluids, and finally, to propose a suitable mechanism behind the enhancements. The study will further analyze the role played by various mechanisms in the enhancement of thermal conductivity. Brownian motion of particles is usually widely reported in the literature. Fluid–particle interaction, liquid layering, clustering, agglomeration of particles, phonon–phonon transport, surface chemical effects, ballistic transport, non-local effect, and near-field radiation will also be addressed.

## 2. Materials and Methods

### 2.1. Synthesis of Ternary-Hybrid Nanoparticles

#### 2.1.1. Preparation of GO-TiO_2_-Ag and rGO-TiO_2_-Ag Nanocomposites

Both GO-TiO_2_-Ag and rGO-TiO_2_-Ag nanocomposites were synthesized using a hydrothermal method [15]. Graphene oxide (GO) of about 0.25 g was suspended in 250 mL of deionized water using an ultrasonic stirring treatment for about 2 h. After that, 10 mL of titanium isopropoxide (TTIP) was dissolved in 10 mL of isopropyl alcohol, and the mixture was added dropwise into the 50 mL of GO suspension. Then, 10 mL of 0.2 M AgNO_3_ was added dropwise into the GO-TTIP mixture and then stirred for approximately 1 h. After stirring for about 1 h, the pH was adjusted to 1.1, and the stirring continued for another 2 h to obtain a uniform solution. The solution was then transferred and heated at 160 °C for 24 h in a Teflon-lined stainless-steel autoclave. After the hydrothermal treatment, the resulting product was first strained and washed using ethanol. Unreacted ions and possible remnants were removed by washing the product with DI water. The resulting GO-TiO_2_-Ag nanocomposite was dried overnight at 80 °C. A similar procedure was repeated to synthesize rGO-TiO_2_-Ag. The difference is that 2 mL of hydrazine and ammonia were added to aid and adjust the pH to neutral. Hydrazine was used to remove the oxygen molecules from graphene oxide sheets and their functional groups. Different hybrid nanofluid samples were prepared by blending various ratios of nanomaterials in DI water. After sonication and stirring, the homogeneous formulation was obtained using a bath-type sonicator (JAC Sonicator 1505, 4 kHz) for about 4 h. Physical monitoring of samples was performed to examine the settling of nanoparticles. 

#### 2.1.2. Materials’ Characterization

A Bruker IFS66/S infrared spectrometer was used in recording Fourier transform infrared spectroscopy (FTIR) spectra. Scanning was completed with a scan number of 16 and a resolution of ±4 cm^−1^ in the range of 4000–400 cm^−1^. This gave the required recorded spectra. A He–Ne laser (model RENISHAW in via Raman Microscope) was used to measure the laser Raman spectroscopy (LRS) at room temperature in the 200–3000 cm^−1^ region through a UV excitation at 325 nm. A field-emission scanning electron microscope (FE-SEM) at 30 kV was used to measure the surface topography. The powder X-ray diffraction (XRD) patterns were taken on a PANanalytical, X’Pert High-Score diffractometer with primary monochromatic high-intensity CuK_α_ radiation (λ = 0.15406 nm).

### 2.2. Synthesis of Hybrid Nanofluids

The challenge in the application of the nanofluid is its preparation and long-term stability. To achieve an even dispersion and obtain the desired characteristics requires the nanofluid to be stable without sedimentation. The ternary-hybrid nanofluids were prepared by dispersing the two types of graphene-based hybrid nanoparticles (GO-TiO_2_-Ag and rGO-TiO_2_-Ag) in the base fluid (molecular biology-grade sterile/DI water). An electronic balance (Sartorius Entris^®^ Analytical balance) was used to weigh the nanoparticles before dispersing them into the base fluid. A stock solution was prepared with 50 mL of deionized water and 2.5 mg of THNp, which constitutes a concentration of 0.05 wt.%. Ultrasound probe sonication was performed on this nanofluid for about 10 min. The nanofluids were then sonicated using a bath sonicator for 4 h until the nanoparticles were dispersed entirely in the deionized water to form a homogenous mixture without any sedimentation. Five levels of serial dilutions from the 0.05 wt.% stock solution were performed.

### 2.3. Thermal Conductivity Measurement

Thermal conductivity measurements on the ternary-hybrid nanofluids were carried out on all the samples using a handheld thermal analyzer, named KD2 pro (Decagon Devices). This thermal analyzer has a transient line heat source method. The heat-flow equation with an exponential integral solution is attached to the device for both single- and dual-needle-type sensors. The device calculates the thermal conductivity and specific heat using a mathematical nonlinear least-squares-inverse procedure to solve differential equations [16].

The samples were immersed in a thermostat bath in equilibrium to minimize free convection. The thermal analyzer was kept isolated to avoid any disturbances and vibrations from other adjacent equipment, which may induce vibrations during the measurements. The transient line source was a single needle (1.3 mm diameter and 60 mm length) used for the thermal conductivity measurements. The sensor needle was calibrated inside the standard liquid (DI water and glycerine, thermal conductivity = 0.633 and 0.285 W/mK, respectively, at ambient temperature) provided by the equipment manufacturer for accuracy of the measuring instrument. Measurements were taken at various temperatures starting at 25 °C, with 5 °C increments until 50 °C. Nonlinear least-squares were performed for exponential integral functions for curve fitting of the measured values. The measurements were iterated four times to yield a well-averaged value and eliminate uncertainty within ±5%.

### 2.4. Dynamic Viscosity Measurement

Dynamic viscosity measurements were performed using Anton Paar’s (GmbH, Austria) MCR302 modular compact rheometer. This rheometer uses a C-PTD200 gauging cell, and the temperature of the measurement sample is maintained with the Peltier system at 1–100 1/s. A CC45 DIN spindle was used in measuring viscosity, shear rate, and shear stress at temperatures ranging from 25 to 50 °C, with an increment of 5 °C. The ternary-hybrid nanofluids were placed in the sample chamber. Measurements of the viscous drag were performed by immersing the double-gap spindle in the sample chamber containing nanofluids with the deflection of the calibrated spring. The viscosity shear strain and shear stress were measured at room temperature, and the equipment’s data logger recorded the data. The accuracy of the rheometer is guaranteed to be within ±1% of the full-scale range. The reproducibility is within ±0.2 rpm for the spindle speed combination. The viscous effect was developed in the ternary-hybrid nanofluids against the spindle rotation. Viscosities of the ternary-hybrid nanofluids were measured at various temperatures ranging from 25 to 50 °C, with 5 °C increments. The viscosity measurements of the hybrid nanofluids showed a linear profile, which indicates that they are Newtonian fluids. The ternary nanofluids’ measurement shows substantially higher viscosities than the base fluids [17].

## 3. Results

### 3.1. Characterization Results

**Figure 1:**
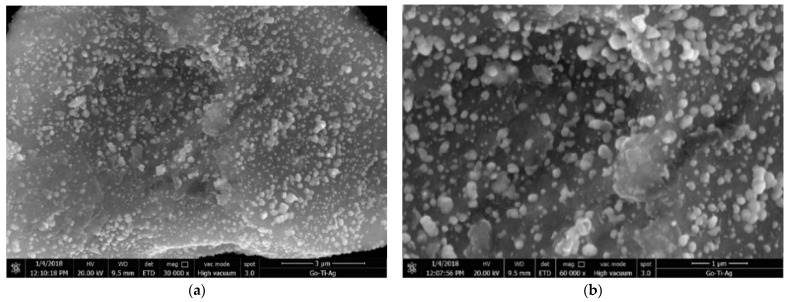
SEM images of GO-TiO_2_-Ag with magnifications of (**a**) 30,000× and (**b**) 60,000×.

**Figure 2:**
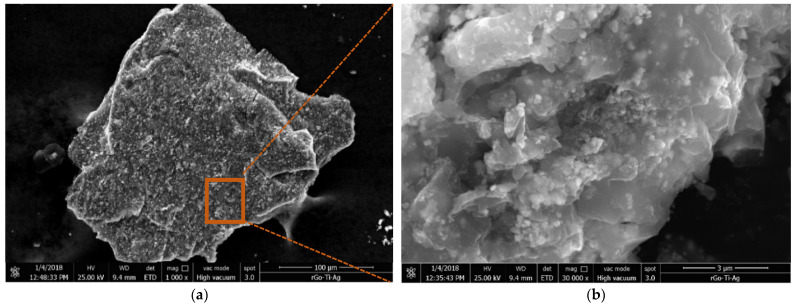
SEM images of rGO-TiO_2_-Ag: SEM magnification of (**a**) 1000× and (**b**) 30,000×.

For the GO-TiO_2_-Ag ternary-hybrid nanoparticle, crumpled and rippled GO sheets were decorated by two types of nanoparticles, the TiO_2_ and the Ag, as shown in Figure 1. However, the rGO-TiO_2_-Ag ternary-hybrid nanoparticle, the rGO, appeared more as layered and wrinkled nanosheets decorated with the TiO_2_ and the Ag [18]. This indicates the success of the deposition of the Ag and the TiO_2_ nanoparticles on the rGO nanosheet (Figure 2).

**Figure 3:**
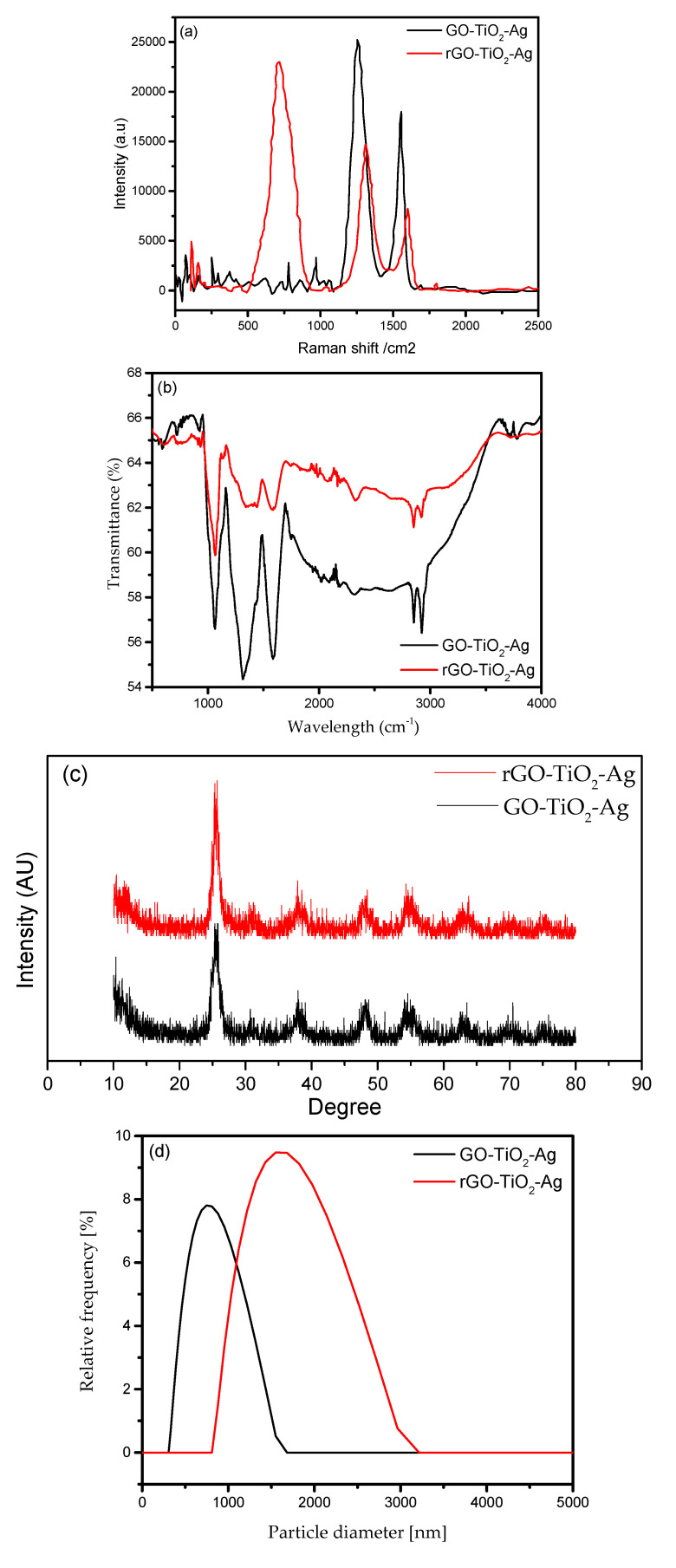

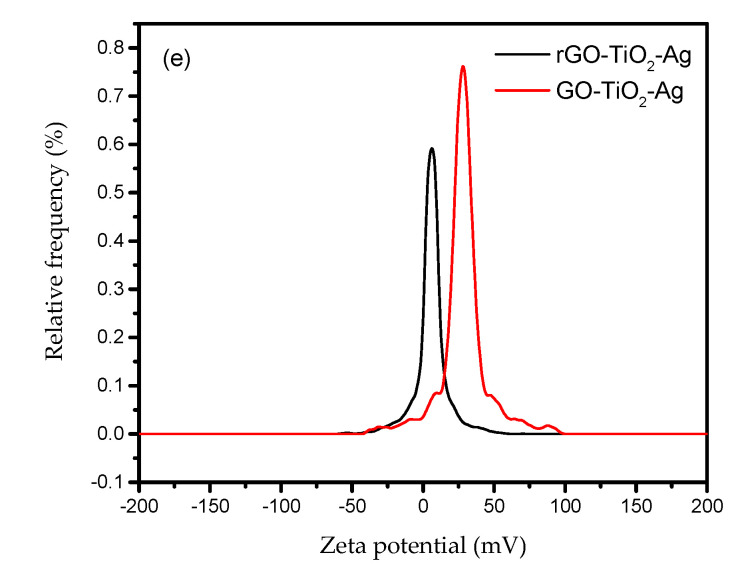
(**a**) Raman spectroscopy of THNp, (**b**) FTIR of THNp, (**c**) XRD of THNp, (**d**) Particle Size Distribution of THNp, (**e**) Zeta potential of THNp.

Figure 3a shows the Raman scattering spectra of the GO-TiO_2_-Ag and rGO-TiO_2_-Ag. The sp^2^-hybridized carbon system’s structural defects are determined mainly by analyzing the D- and G-bands in the Raman spectra. These bands represent the presence of first-order scattering of the E_2g_ phonons of sp^2^ carbon atoms. The peaks of the D- and G-bands are presented alongside 637 cm^−1^, which is the E_g_ peaks. This is due to the symmetric stretching vibration of the O–Ti–O bonds in both nanocomposites [19]. Nonetheless, the peak intensity ratio, I_D_/I_G_, of rGO-TiO_2_-Ag increased to 1.41 compared to the GO-TiO_2_-Ag (0.96). This may be mainly due to higher defects in its graphitic domains during the chemical reduction of the GO. 

The FTIR spectrum of the nanocomposites (GO-TiO_2_-Ag and rGO-TiO_2_-Ag) are shown in Figure 3b. The FTIR spectrum of the GO-TiO_2_-Ag possesses strong absorption bands at 1723, 1620, and 1223 cm^−1^, corresponding to stretching vibration of C=O, C-C, and C-O, respectively. There are essential oxygen molecules present in the GO alongside a broad hydroxyl peak at 3300 cm^−1^. The peaks at 1620 and 1223 cm^−1^, along with a smaller peak at 596 cm^−1^, may attribute to the stretching vibration of the (NH) C=O group of silver nanoparticles [20]. Moreover, the peak at 1000 cm^−1^ in composites is attributed to the Ti-O=Ti and Ti-O-C groups. However, some peaks are still present in the rGO-TiO_2_-Ag spectra, but the oxygen peak appears at a lower intensity due to the exclusion of oxygen molecules during the reduction process [21]. Therefore, the essential peak of Ag and TiO_2_ is still present in the FTIR of rGO-TiO_2_-Ag, which indicates the successful manifestation of the silver and titanium nanoparticles on the rGO. 

The crystallographic structures of the synthesized GO-TiO_2_-Ag and rGO-TiO_2_-Ag nanocomposites were gauged with XRD. The XRD spectra are as shown in Figure 3c. It is a series of reflections that peaks at 24.5° (101), 38.9° (004), 49.5° (200), 55.4° (105), and 64.2° (204), corresponding to the anatase phase of titanium [22]. Moreover, the peaks at 70.5° (220) and 79.5° (311) belong to silver nanoparticles [23]. The diffraction peaks of the GO-TiO_2_-Ag are not distinguishable with the rGO-TiO_2_-Ag due to the lower crystallinity degree of the GO and the rGO compared to the TiO_2_, which resulted in the shielding of the peaks [24].

The particle size distribution of the THNp in the nanofluids was carried out using the dynamic light scattering method. The measurements were performed using the Anton Paar Particle Size Analyzer PSA 990. The scattering ranged from 0.2 to 500 μm, and it has an accuracy of <3% with less than a minute of measuring time. The ternary-hybrid nanofluids were filled in the vial and placed in the analyzer. Graphene-based hybrid nanofluids are usually well-dispersed colloids since they are polar and hydrophilic. Figure 3d shows the aggregate size of the ternary hybrids. The GO-based ternary hybrid has particle size peaks at 750 nm, while the rGO-based ternary hybrid is at the peak of 1750 nm. The dispersion stability of the nanofluids and surface charge at the solid–liquid interface is measured in terms of zeta potential. The zeta potential of the THNp-based nanofluids was measured using the Anton Paar Electrokinetic Analyzer SurPASS 3. The electrostatic repulsion forces of the suspended nanoparticles and the zeta potential are directly related. The attraction forces for the precipitation should be less than the electrostatic repulsion force [25]. The absolute zeta potential of 30 mV is considered suitable for the stability of a solid in liquid colloids, which has low ionic strength. Figure 3e shows that the hybrid nanoparticles’ peak ranges from 25 to 35 mV, within a stable range. On the other hand, a lower zeta potential of about 20 mV is considered unstable dispersion.

### 3.2. Thermal Conductivity Measurements of Synthesized GO-TiO_2_-Ag and rGO-TiO_2_-Ag

#### 3.2.1. Effect of Concentration

**Figure 4:**
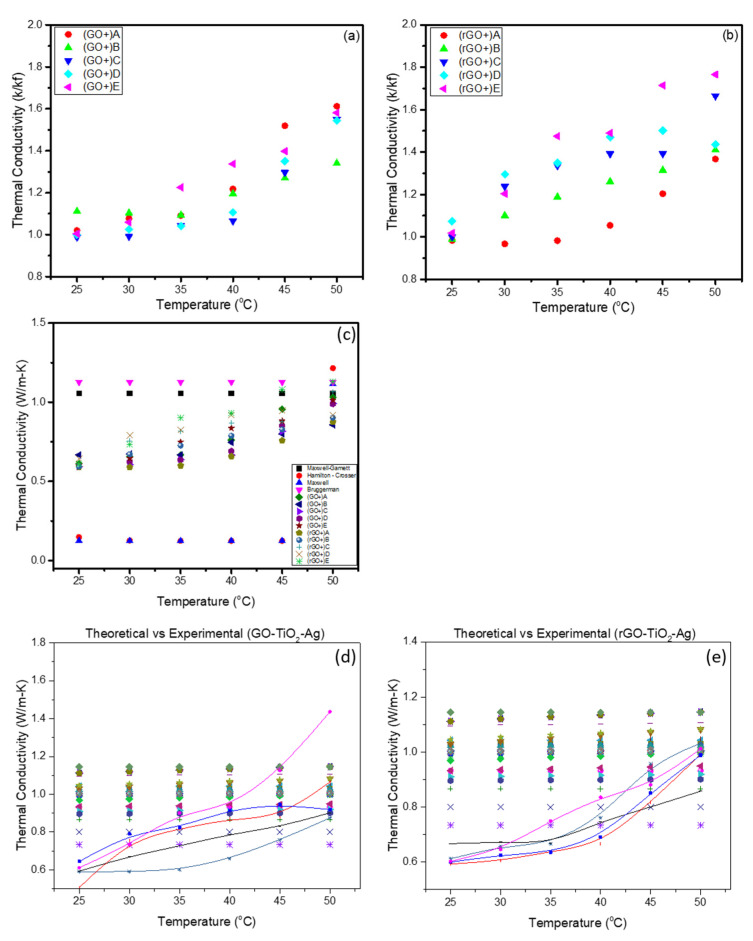
(**a**) Thermal conductivity of THNp GO-TiO_2_-Ag. (**b**) rGO-TiO_2_-Ag at various temperatures (where A (5 × 10−1 wt.%), B (5 × 10−2 wt.%), C (5 × 10−3 wt.%), D (5 × 10−4 wt.%), and E (5 × 10−5 wt.%) are serial dilutions). (**c**) Comparison of experimental values of GO-TiO_2_-Ag nanofluids with classical theoretical models. (**d**,**e**) Comparison of experimental values of GO-TiO_2_-Ag and rGO-TiO_2_-Ag nanofluids with the existing hybrid empirical correlations respectively. The legend of 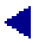, 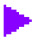, 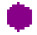, 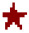, and 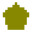 are the data from Afrand et al. [29] hybrid nanoparticle, while 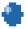, 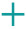, 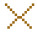, 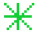, and 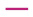 are Nabil et al. [9] data. 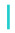, 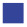, 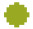, 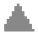, and 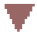 are Esfahani et al. [7] data, while 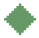, 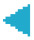, 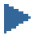, 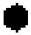, and 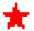 are Hemmat et al. [30] data. 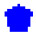, 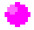, 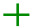, 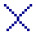, and 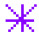 are Hemmat et al. [31] data, also 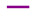, 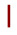, 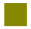, 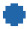, and 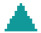 Hemmat et al. [32] data as well as 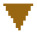, 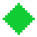, 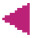, 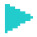, and 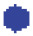 Hemmat et al. [33]. 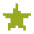, 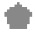, 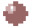, 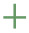, and 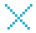 are Rostamian et al. [34] data. 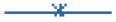, 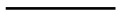, 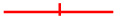, 
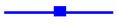
, and 
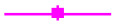
 are the data of rGO-TiO_2_-Ag at concentration A (5 × 10−1 wt.%), B (5 × 10−2 wt.%), C (5 × 10−3 wt.%), D (5 × 10−4 wt.%), and E (5 × 10−5 wt.%), respectively.

The thermal conductivity of ternary-hybrid nanofluids with different volumetric concentrations ranging from 0.05 wt.% to 0.0005 wt.% was measured. The results are presented in Figure 4a,b. The measurements were carried out for the ternary-hybrid nanofluids with temperatures ranging from 25 to 50 °C, and with increments of 5 °C. It can be observed that the heat transfer characteristics of the nanofluids increase linearly with the increase in volume fraction/concentration. The carbon-based nanofluids’ thermal conductivity is generally said to be higher, as reported by many research observations [26]. The thermal conductivity of the nanofluids is usually higher than the standard base fluids. This increase is attributed to the thermal conductivity of the nanoparticles in the nanofluids.

The thermal conductivity also increases significantly with the increase of the temperature of the fluid. Significant enhancements in thermal conductivity can be observed from the plots in all samples shown in Figure 4a,b. The duration of sonication plays a vital role in the increase of the thermal conductivity of the nanofluid. It was reported that the particle size could be controlled by applying ultra-sonication. The surface area in the nanofluid is increased by making the particle shape and size uniform. This will increase the thermal conductivity of the fluid. A lower concentration of nanoparticles is chosen to prevent the undesirable increase in the viscosity of the nanofluids.

The measurements show that the thermal conductivity increases even with a very low concentration of THNp. The thermal conductivity enhancement at 25 °C is about 10%, whereas the enhancement at 50 °C is about 60%. This enhancement is good considering such a small volume fraction. The substantial increment in the thermal conductivity can be attributed to the greater surface area of the THNp. The concentration of nanoparticles has a direct impact on the thermal conductivity of the nanofluids. For instance, heat transfer occurs at the surface of the particles; therefore, nanofluids with higher concentrations show higher thermal conductivity. This is due to the effect of Brownian motion on the surface of the particles. It can be observed from the figures that the nanofluids with a lower concentration have lower thermal conductivity, whereas the nanofluids with a higher concentration have slightly higher thermal conductivity. It may be due to the surface to volume ratio being 1000 times smaller for particles of 10 nm in diameter in every serially diluted nanofluid. 

#### 3.2.2. Effect of Temperature

The nanofluids are used in several applications where a general change of temperature is expected. Many studies establish that thermal conductivity and fluid temperature are directly related [27]. Thus, thermal conductivity enhances with the increase in temperature of the fluid. It is worth noting that the enhancement of thermal conductivity in nanofluids is the function of the nanoparticles and temperature. In this study, the nanofluid temperature is increased from 25 to 50 °C, and the thermal conductivity of the standard base fluid added with ternary-hybrid nanoparticles steadily increased. The enhancement of thermal conductivity is because of the increase in temperature due to the materials’ natural thermal conductivity phenomena. Majority of nanomaterials’ thermal conductivity increases with the rise in temperature that transfers higher energy between the particles in the standard base fluid. In addition, the increase in the fluid’s temperature increases the molecular movements, which enhance energy transfer. If there is any change in the nanofluids’ dispersion characteristics, it can be detrimental to the heat transfer process of the nanofluids. The nonlinear thermal conductivity enhancement process was observed by Wen and Ding [3] for CNT/water nanofluid. They observed an increase in the thermal conductivity due to higher temperatures and a smaller particle size, which was higher than that of the lower temperature and larger particle size. The correlations based on experimental data of hybrid thermal conductivity models with water as a base fluid are summarized in Table 1, and the plots are illustrated in Figure 4c,d. The plots are divided based on the THNp used (GO-TiO_2_-Ag and rGO-TiO_2_-Ag). Existing empirical models predict the thermal conductivity of a particular hybrid nanoparticle within certain limits of concentration and temperature. It can be observed from the plots that there are discrepancies in the data based on the models proposed by various researchers as they are derived for fitting experimental results for the hybrid nanofluids. It can also be seen that all the hybrid nanoparticles reported are bi-hybrid nanoparticles, and the range of the concentrations is highly varying from the experimental results obtained in this study. This study deals with THNp of very low concentration, and none of the reported empirical correlations are even close. The measured temperature of 25 to 50 °C shows that the experimental data are in a nonlinear trend for the thermal conductivity of the nanofluids. There are very few empirical correlations with water as the base fluid, and other empirical correlations are mostly either ethylene glycol-water (EG-water) or EG alone. Hence, the idea of applying the existing empirical correlations to our novel THNf remains inconclusive. Significant differences can be observed in the thermal conductivity. The hybrid nanofluids with different base fluids are linear in the case of water and nonlinear in EG. This incompatibility of the previous studies’ empirical correlations with the THNf calls for developing a new correlation, which is planned in the near future, and to compare with other classical physics-based models, as has been reported by Eapen et al. [28].

### 3.3. Mechanisms Influencing the Thermal Conductivity Enhancements

As shown in Figure 4a,b, a combination of several factors are known to contribute to the enhanced thermal conductivity effect in hybrid nanofluids. We list some fundamental mechanisms that probably influence our results.

In our case, the fluid’s ternary-hybrid nanoparticles collide due to Brownian motion, generating energy and subsequently dissipating through the surrounding liquid. Theoretically, a higher concentration of nanoparticles in the fluids should lead to higher thermal conductivity, but higher concentrations of particles tend to have stability issues, leading to nanoparticles’ sedimentation. From the measurements, it was observed that THNp in the nanofluids enhances the thermal conductivity in a linear increment, where the thermal conductivity of the THNf increases with temperature. Usually, smaller particles have higher vibrations, which increase the absorption of the quantized thermal energy by the atoms. According to the Stokes–Einstein formula, the Brownian motion can be represented as:

(1) $$D=\frac{K_{B}T}{3\pi \eta d}$$

where *D* is the particle diffusion coefficient, *K_B_* is the Boltzmann constant, *η* is the fluid’s viscosity, and d is the particle’s diameter. The overall effect of Brownian motion on the thermal conductivity in the THNf is the timescale of particle motion concerning the diffusion of heat energy in the THNf. Brownian motion leads to thermal conductivity on a microscale or nanoscale level, and it cannot be neglected. It has a more significant say in the conduction of thermal energy compared to the thermal diffusivity of the base fluids [35]. The suspended particles with higher dialectic forces are typically charged, leading to repulsive force between the particles that inhibits agglomerations. The continuous interaction between the molecules and particles results in the transfer of heat. At the nanoscale level, the Brownian motion of nanoparticles governs the thermal behavior of the nanofluids. The contribution of Brownian motion in the thermal conductivity in the nanofluids can be in two ways. First, the stochastic motion will be more significant for higher temperatures. Studies have indicated that the particles’ Brownian motion will only result in milder thermal conductivity, which is negligible in lower temperatures. They also proposed a threshold temperature corresponding to the size of the nanoparticle. The Brownian motion of particles and the clustering of nanoparticles are directly proportional to the change in the nanofluid temperature, which increases the thermal conductivity. Although our measurements are below 50 °C due to the equipment limitation, we hypothesize that the effect of Brownian motion could be prominent at higher temperatures. 

Clustering is another crucial mechanism behind enhancing thermal conductivity in nanofluids. It is the aggregation of particles resulting from interacting forces. In the case of ternary hybrids, graphene oxide, silver, and titanium oxide nanoparticles have been purposely clustered. Clustering creates a path of lower thermal resistance, leading to enhanced thermal transfers [36] due to the shorter distance between the graphene oxide and silver-titanium oxide particles, which can be observed from the SEM graphs in Figure 1 and Figure 2. However, if the clustering occurs with more nanoparticles, it leads to particles’ sedimentation in the fluid. A higher number of clusters are usually typical in nanofluids with higher concentrations, leading to sedimentation. On the other hand, these aggregations are against particles’ dispersal, triggering the particles to be packed together to form a solid zone in colloids. The thermal energy in a solid crystalline lattice tends to move faster than in any other liquid. Hence, the THNp increases the effective thermal conductivity in a colloidal solution. Recent studies have shown that aggregation enhances the thermal conductivity of nanofluids [37]. 

**Figure 5:**
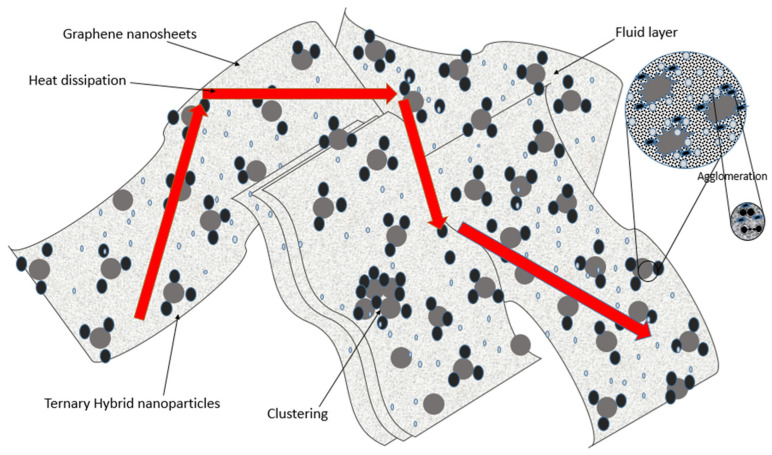
Schematic of the proposed thermal conductivity mechanism of THNp in the nanofluids.

The layers of liquids nearest to the THNp’s surface tend to form structures in nanometers that imitate and behave as solids at the solid–liquid interface, where heat transfer occurs. Graphene oxide has a high thermal conductivity, which enhances the thermal conductivity of the nanofluids. In the THNf, graphene oxide and silver and titanium oxide form a layer around the particles. The fluid particles in the nanoscale have a similar pattern of forming layers at the interactions with THNp. These layers play a vital role in transferring heat from solid THNp to adjacent liquid layers [38]. Since the nanoparticles have a higher surface area, the interaction to these liquid layers is higher, and therefore the thermal conductivity is also higher. The thickness of the fluid layers would be on the nanometer scale, but it plays a significant role in transferring heat from solid THNps to the adjacent layer of the liquid interface. The thermal conductivity of the interfacial layer would be almost similar to that of the nanoparticles. Studies and models have shown that although the liquid layer plays a vital role in the thermal conductivity, it may not be the dominant factor in the overall increase of thermal conductivity in the nanofluids. A schematic of liquid layering is presented in Figure 5. Liquid layering will be dissimilar, and their interaction would be different for different shapes of nanoparticles. For example, spherical nanoparticles will have the spherical shape of liquid layers, while other shapes will be different [39]. 

The phonons are the vibrations that play a crucial role in the nanoparticle’s thermal conductivity in the fluid. The phonon density is higher in the hotter region than the colder regions, leading to thermal conduction from the hot to the cold region in the fluid. In the solid–liquid mixture, as in THNfs, it is assumed that thermal conduction occurs in both solid and liquid materials present in the fluids. Phonons carry the heat energy in the THNf on a nanoscale from one particle to another as carriers by quantization of lattice vibrations. Phonons can be treated as a particle or a wave in the THNf. They demonstrate similar features, which may be the main reason for naming it transport rather than transfer by which heat is transported in a dynamic particle system. The faster movement of phonons increases the thermal conductivity in the THNf. The average distance between the phonon–phonon collisions distinguishes the heat transfer regime from the particle’s heat transfer. At the same time, on the other side, when the phonons do not collide, the transfer rate of heat is higher since it does not collide and scatter.

Thermal conductivity and rheological measurements of nanofluids with metal oxides for a concentration of >1% were studied by many researchers. However, minimal experimental data are available for effective thermal conductivity and viscosities of the nanofluids made of pure metallic nanoparticles at very low volume concentrations (<1%) and higher temperatures. The present study is very significant in fulfilling this gap [17]. THNf has high complexity in its structure after synthesis since it has three different types of nanoparticles attached by covalent bonds and water molecules. Hence, several parameters and mechanisms are responsible for the heat transfer in the THNf, such as atomic forces, inter-particle forces, and other external factors, along with surface forces. Interfacial resistance between the particles in the THNf may also increase the thermal conductivity. The ternary-hybrid nanoparticles’ inherent thermal conductivity may be due to the advantage of forming clusters of the THNp by concomitant degradation of convection. As shown in the suggestive schematic in Figure 5, the graphene layers or flakes are stacked. The titanium and silver nanoparticles agglomerate on the flakes. The percolation networks with aniosphic thermal conductivity of the nanoparticles result in higher thermal conductivity. 

Other mechanisms that may contribute to higher thermal conductivity in nanofluids could be phase change and surface chemical effects. Heat conduction in solid materials is more complicated than in liquids. Few other mechanisms debated by researchers include the diffusion of thermal energy (thermodiffusion/thermomigration/thermophoresis) in a faster fluid medium than translational movements [40]. The other possible phenomenon is the micro-convection effect, which is defined as convectional heat transfer due to the micro-mixing impact and the translational displacement because of the fast rotation of the THNp in the nanofluids. There is no doubt that the nanoscale rotation of particles will increase the movement of the fluid molecules when the temperature is increased, leading to an increase in the nanofluids’ thermal conductivity. The thermal conductivity may also be affected by the particle density, size and shape, type of base fluid, concentration of the particle, pH, effect of sonication, effect of particle aspect ratio, the effect of particle material, and nano-inclusions [41]. In addition, the crystalline nature of the THNp and its functionalized structure may act as extended solid surfaces in the fluid, which may also enhance the thermal conductivity.

### 3.4. Viscosity Measurements of GO-TiO_2_-Ag and rGO-TiO_2_-Ag

#### 3.4.1. Viscosity vs. Temperature

**Figure 6:**
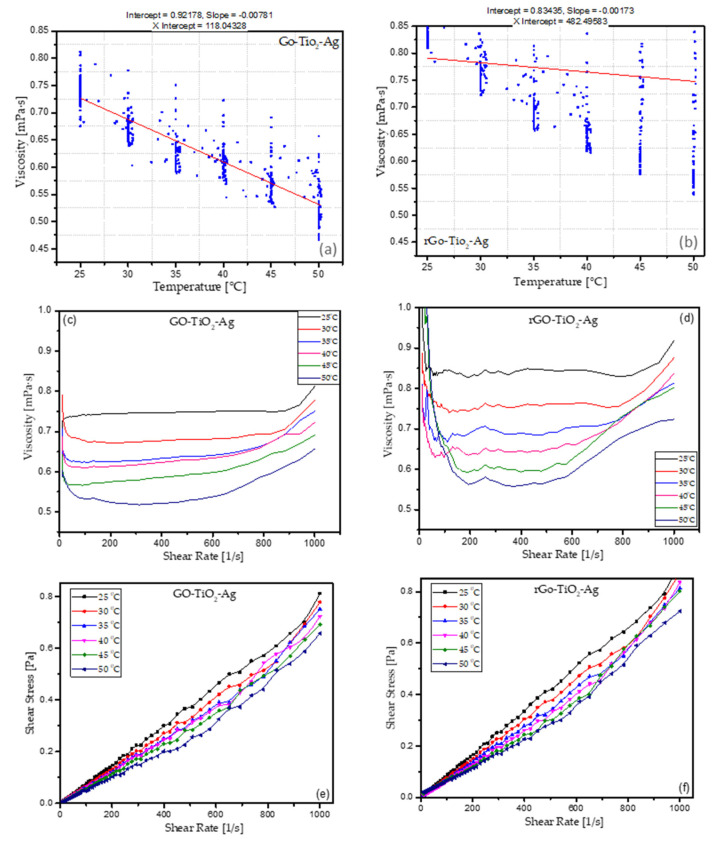
(**a**) Viscosity of THNp GO-TiO_2_-Ag, (**b**) viscosity of THNp rGO-TiO_2_-Ag, (**c**) viscosity vs. the shear rate of THNp GO-TiO_2_-Ag, (**d**) viscosity vs. the shear rate of THNp rGO-TiO_2_-Ag, (**e**) shear stress vs. the shear rate of THNp GO-TiO_2_-Ag, and (**f**) shear stress vs. the shear rate of THNp rGO-TiO_2_-Ag.

Rheological measurements of both the THNp (GO-TiO_2_-Ag and rGO-TiO_2_-Ag)-based nanofluids were performed at a concentration of 0.05 wt.% (concentration A—stock solution). It can be observed that viscosity is lower at higher temperatures. The hybrid nanoparticle shows signs of Newtonian individuality in all the temperature ranges that were measured. The rheological behavior of hybrid nanofluids is imperative to its application. The viscosity profile is advantageous for the hybrid nanofluid’s application even in conditions of variable shear rate. The viscosity profile of the ternary-hybrid nanofluids is presented in Figure 6a,b. The viscosity decreases with the increase in the temperature of the fluid. The inter-particle and intermolecular adhesion forces weaken with the increase of temperature, thereby reducing the viscosity. When the kinetic energy of the molecules increases with the temperature rise, the particle closest reduces the contact. This gap reduction decreases the intermolecular forces, leading to a decrease in the viscosity of the nanofluids [42]. The shear stress is linearly dependent on the shear rate, which shows the Newtonian behavior of the ternary-hybrid nanofluids. An increase in the line’s slope shows the increase in volumetric concentration and volumetric particle fraction of the ternary-hybrid nanofluids. The temperature dependency behavior of the ternary-hybrid nanofluid’s viscosity is evident based on Figure 6a,b. A sharp decrease in the viscosity was noticed with the increase in the temperature of the nanofluid. The viscosity of the nanofluids with various metallic, oxide, and carbon-based nanoparticles increases with particle loading or an increase in the concentration of particles. It decreases with the increase in temperature, as reported by many researchers. Many research studies have illustrated that the viscosity increased with the increased concentration volume [43]. The particle size also plays a significant role in the increase in viscosity. Viscosity measurements by Murshed et al. [44] indicated an increase in viscosity with the increase in the volumetric loading of nanoparticles. At the same time, Lee et al. [45] reported a decrease in viscosity with the increased temperature. 

#### 3.4.2. Viscosity vs. Shear Rate

Rheological measurements of the hybrid nanofluids were carried out by varying the shear rate of the nanofluid samples under constant temperature. The shear stress in any fluid helps to determine if the fluid is Newtonian or non-Newtonian. Figure 6c,d illustrate the measured shear stress vs. the shear rate exerted on the respective sample of the ternary-hybrid nanofluids. The measurements were carried out at temperatures ranging from 25 to 50 °C with an increment of 5 °C for shear rates of 10 and 1000 1/s. It can be observed that the shear stress is linearly dependent on the shear strain for the ternary-hybrid nanofluids. The viscosity of the nanofluids is generally higher than the base fluids. The slope of the line increases linearly with the rise in the volumetric concentration. The increase in shear stress increases the viscosity and volumetric concentration. The increase in the shear rate increases the particle–particle interactions, which leads to a decrease in viscosity. It is also influenced by the concentration and the type of nanoparticles. The increase in the nanoparticle concentration leads to an increase in the nanofluids’ viscosity, as reported by many researchers [4]. The aggregation of nanoparticles at a higher concentration can be one of the factors that influence the viscosity. This aggregation leads to the enhancement of internal shear stress in the nanofluids. Therefore, a higher force is required for dissipation of the substantial dispersion component, which leads to an increase in the nanofluids’ viscosity. Micro-aggregation of nanoparticles leads to a higher volume fraction, which increases the viscosity. Figure 6e and f show that the shear rate is a function of shear stress, which means that the shear stress of the ternary-hybrid nanofluids is directly dependent on the shear stress of the nanofluids when the viscosity is constant. As the temperature of the nanofluids increases, the shear stress and the shear rate increase, which indicate the Newtonian behavior of the ternary-hybrid nanofluids. The shear viscosity at 25 °C is approximately double that at 50 °C, indicating an intense temperature-dependent nature of the ternary-hybrid nanofluids. The rheological behavior was further studied with the increase in temperature of the nanofluid. The results show similar Newtonian behavior with a further increase in temperature of the ternary-hybrid nanofluids. Similarly, Newtonian behaviors were reported by many other researchers for the hybrid nanofluids based on iron and copper nanoparticles [46], Fe_3_O_4_-MWCNTs/EG hybrid nanofluid [47], SWCNTs–CuO [34,48], and Al_2_O_3_-SiO_2_/PAG [49], etc. As the temperature increases, the ternary-hybrid nanofluids behave as shear-thinning fluids that lowers the shear viscosity and increases the shear rate. The decrease of viscosity with the increase of temperature is a common phenomenon. However, with a combination of three types of nanoparticles decorated on each other, the nanoparticles in the fluids may behave differently. The GO-based nanofluids, in contrast with the rGO-based nanofluids, have a more significant change in viscosity. We can observe that the deposition of silver and titanium oxide into the reduced graphene oxide is much more compact than the GO-based THNp. This may affect the viscosity, which can be observed from Figure 6a,b. There are various viscosity correlations in the literature for hybrid nanofluids that may explain the behavior of ternary-hybrid nanofluids in base fluids. A comprehensive study of the ternary-hybrid nanofluids and their serial dilutions with available correlations will be carried out in the future.

The measurements show a similar trend of shear-thinning for both the ternary-hybrid nanofluids. There is a fair agreement and similarity between the two nanofluids presented in the figures, repeatedly showing the measurements and stability of the temperatures. The shearing effects on the nanoparticles in the fluid can affect the particle orientation, which causes aggregated particles’ disintegration. Shear-thinning behavior can occur due to various situations and stresses applied to the fluid, reducing viscosity. When the nanofluids’ temperature increases, the Brownian motion increases, along with its average speed and the thermal movement of the molecules. This process weakens the adhesion forces and the intermolecular interaction between the molecules. The effects of the nanoparticle size, shape, pH, temperature, and volumetric concentrations may increase or decrease the ternary-hybrid nanofluids’ viscosity. Viscosity change may also be dependent on the type of base fluids used, the particle volume fraction, the particle size distribution, the shear rate, the surfactants, the dispersion techniques, and particle aggregation. Clustering is another factor that influences the viscosity of nanofluids. This means that very few nanoparticles collide with each other. In contrast, other nanoparticles may have an interfacial layer developed around them in various studies and of the working hypotheses. The findings and their implications should be discussed in the broadest context possible. Further viscosity studies on the serial dilutions and their effect of viscosity will be carried out, and future research directions may also be highlighted.

## 4. Conclusions

Two novel metal-metal oxide-carbon combinations forming ternary-hybrid nanoparticles were synthesized and thoroughly characterized. The results show that by adding 0.01 wt.% of GO-TiO_2_-Ag and rGO-TiO_2_-Ag, the thermal conductivity of H_2_O increased by 66% and 83%, respectively. Various mechanisms, such as Brownian motion, liquid layering, phonon transport, particle clustering, and the percolation effect, could be contributing to the observed enhancement. Comparison of the experimental data with the existing empirical correlations and classical theoretical models, such as Maxwell’s, Hamilton–Crosser, Maxwell–Gamet, and Bruggerman, showed that those models could not predict the effective thermal conductivity of the ternary-hybrid nanofluids used in the study. Hence, it calls for the development of new models on metal-metal oxide-carbon combinations. Rheological investigation of the nanofluids showed that the nanofluids tended to display Newtonian and non-Newtonian behavior based on different parameters. In most cases, the viscosity decreased with the nanofluids’ temperature, as established in the existing literature. The change in viscosity with temperature of ternary-hybrid nanofluids could be due to the agglomeration of nanoparticles. It is noteworthy that both the viscosity and thermal conductivity depend on the concentration of nanoparticles.

## Figures and Tables

**Table 1:** Empirical correlations for the thermal conductivity of water-based hybrid nanoparticles.

Nanoparticle	Base Fluid	Empirical Correlation	Range	Authors
SiO_2_-TiO_2_	water and EG (60:40)	$K_{eff}=\frac{K_{nf}}{K_{bf}}=1.17{\left(1+R\right)}^{-0.1151}{\left(\frac{T}{80}\right)}^{0.0437}$	1:4 to 4:1 30 to 80 °C	Afrand et al. [29]
SiO_2_-TiO_2_	water and EG (60:40)	$\frac{K_{nf}}{K_{bf}}={\left(1+\frac{\phi }{100}\right)}^{5.5}{\left(\frac{T}{80}\right)}^{0.01}$	0.5% to 3% 30 to 80 °C	Nabil et al. [9]
ZnO-Ag	water	$\frac{K_{nf}}{K_{bf}}=1+0.0008794\phi^{0.5899}T^{1.345}$	0.125% to 2% 25 to 50 °C	Esfahani et al. [7]
Ag-MgO	water	$\frac{K_{nf}}{K_{bf}}=\frac{0.1747X10^{5}+\phi_{p}}{0.1747X10^{5}-0.1498X10^{6}\phi_{p}+0.11117X10^{7}\phi_{p}^{2}+0.1997X10^{8}\phi_{p}^{3}}$	0 to 3%	Hemmat et al. [30]
DWCNTs-ZnO	water and EG (60:40)	$\frac{K_{nf}}{K_{bf}}=0.0288X\ln\left(\phi \right)+1.085e^{\left(0.001351T+0.13\phi^{2}\right)}$	0.025% to 1% 30 to 50 °C	Hemmat et al. [31]
ZnO-MWCNTs	water and EG (50:50)	$\frac{K_{nf}}{K_{bf}}=1.024+0.5988\phi^{0.6029}\exp\left(\frac{\phi }{T}\right)-\frac{8.059\phi T^{0.2}+2.24}{6.025\phi^{0.02}+T}$	0.02% to 1% 30 to 50 °C	Hemmat et al. [32]
SWCNTs-ZnO (30:70)	water and EG	$\frac{K_{nf}}{K_{bf}}=0.8707+8.883X10^{-4}XT\phi +4.435X10^{-3}\phi^{0.252}T+0.179\phi^{0.179}\exp\left(0.09624\phi^{2}\right)$	0.05% to 1.6% 26 to 50 °C	Hemmat et al. [33]
SWCNTs-CuO	water and EG (60:40)	$\frac{K_{nf}}{K_{bf}}=1+\left(0.04056X\left(T\phi \right)\right)-\left(0.003255X{\left(T\phi \right)}^{2}\right)+\left(0.0001181X{\left(T\phi \right)}^{3}\right)-\left(0.000001431X{\left(T\phi \right)}^{4}\right)$	0.02% to 0.75% 20 to 50 °C	Rostamian et al. [34]

## Data Availability

All data are provided within this manuscript.

## References

[1] Chrystal G. (1882). A Treatise on Electricity and Magnetism an Elementary Treatise on Electricity.

[2] Baig N., Kammakakam I., Falath W. (2021). Nanomaterials: A review of synthesis methods, properties, recent progress, and challenges. Mater. Adv..

[3] Wen D., Ding Y. (2004). Experimental investigation into convective heat transfer of nanofluids at the entrance region under laminar flow conditions. Int. J. Heat Mass Transf..

[4] Sajid M.U., Ali H.M. (2018). Thermal conductivity of hybrid nanofluids: A critical review. Int. J. Heat Mass Transf..

[5] Motevasel M., Nazar A.R.S., Jamialahmadi M. (2018). The effect of nanoparticles aggregation on the thermal conductivity of nanofluids at very low concentrations: Experimental and theoretical evaluations. Heat Mass Transf. Und Stoffuebertragung.

[6] Esfe M.H., Alirezaie A., Rejvani M. (2017). An applicable study on the thermal conductivity of SWCNT-MgO hybrid nanofluid and price-performance analysis for energy management. Appl. Therm. Eng..

[7] Esfahani N.N., Toghraie D., Afrand M. (2018). A new correlation for predicting the thermal conductivity of ZnO–Ag (50%–50%)/water hybrid nanofluid: An experimental study. Powder Technol..

[8] Akhgar A., Toghraie D. (2018). An experimental study on the stability and thermal conductivity of water-ethylene glycol/TiO_2_-MWCNTs hybrid nanofluid: Developing a new correlation. Powder Technol..

[9] Nabil M.F., Azmi W.H., Hamid K.A., Mamat R., Hagos F.Y. (2017). An experimental study on the thermal conductivity and dynamic viscosity of TiO_2_-SiO_2_ nanofluids in water: Ethylene glycol mixture. Int. Commun. Heat Mass Transf..

[10] Arshad A., Jabbal M., Yan Y., Reay D. (2019). A review on graphene based nanofluids: Preparation, characterization and applications. J. Mol. Liq..

[11] Sharma A.K., Tiwari A.K., Dixit A.R. (2016). Rheological behaviour of nanofluids: A review. Renew. Sustain. Energy Rev..

[12] Deepa C., Rajeshkumar L., Ramesh M. (2022). Preparation, synthesis, properties and characterization of graphene-based 2D nano-materials for biosensors and bioelectronics. J. Mater. Res. Technol..

[13] Thamilselvi V., Radha K.V. (2017). A Review on the Diverse Application of Silver Nanoparticle. IOSR J. Pharm..

[14] Naphon P., Thongjing C. (2014). Pool boiling heat transfer characteristics of refrigerant-nanoparticle mixtures. Int. Commun. Heat Mass Transf..

[15] Perera S.D., Mariano P.G., Vu K., Nour N., Seitz O., Chabal Y., Balkus K.J. (2012). Hydrothermal synthesis of graphene-TiO_2_ nanotube composites with enhanced photocatalytic activity. ACS Catal..

[16] Bobrowska A., Jagoda E., Domonik A., Ryżyński G. (2022). Thermomechanical properties of detrital limestone from the Nowe Brusno town (Poland). Resour. Policy.

[17] Godson L., Raja B., Lal D.M., Wongwises S. (2010). Experimental investigation on the thermal conductivity and viscosity of silver-deionized water nanofluid. Exp. Heat Transf..

[18] Leong K.H., Sim L.C., Bahnemann D., Jang M., Ibrahim S., Saravanan P. (2015). Reduced graphene oxide and Ag wrapped TiO_2_ photocatalyst for enhanced visible light photocatalysis. APL Mater..

[19] Choi B.-K., Choi W.-K., Park S.-J., Seo M.-K. (2018). One-Pot Synthesis of Ag-TiO_2_/Nitrogen-Doped Graphene Oxide Nanocomposites and Its Photocatalytic Degradation of Methylene Blue. J. Nanosci. Nanotechnol..

[20] Gurunathan S., Han J.W., Park J.H., Kim E., Choi Y.J., Kwon D.N., Kim J.H. (2015). Reduced graphene oxide-silver nanoparticle nanocomposite: A potential anticancer nanotherapy. Int. J. Nanomed..

[21] Zhang H., Wang X., Li N., Xia J., Meng Q., Ding J., Lu J. (2018). Synthesis and characterization of TiO_2_/graphene oxide nanocomposites for photoreduction of heavy metal ions in reverse osmosis concentrate. RSC Adv..

[22] Thomas R.T., Rasheed P.A., Sandhyarani N. (2014). Synthesis of nanotitania decorated few-layer graphene for enhanced visible light driven photocatalysis. J. Colloid Interface Sci..

[23] Fan B., Guo H., Shi J., Shi C., Jia Y., Wang H., Chen D., Yang Y., Lu H., Xu H. (2016). Facile one-pot preparation of silver/reduced graphene oxide nanocomposite for cancer photodynamic and photothermal therapy. J. Nanosci. Nanotechnol..

[24] Liu H., Duan C., Su X., Dong X., Huang Z., Shen W., Zhu Z. (2014). A hemoglobin encapsulated titania nanosheet modified reduced graphene oxide nanocomposite as a mediator-free biosensor. Sens. Actuators B Chem..

[25] Ghadimi A., Metselaar I.H. (2013). The influence of surfactant and ultrasonic processing on improvement of stability, thermal conductivity and viscosity of titania nanofluid. Exp. Therm. Fluid Sci..

[26] Jabbari F., Rajabpour A., Saedodin S. (2017). Thermal conductivity and viscosity of nanofluids: A review of recent molecular dynamics studies. Chem. Eng. Sci..

[27] Colangelo G., Favale E., Miglietta P., Milanese M., de Risi A. (2016). Thermal conductivity, viscosity and stability of Al_2_O_3_-diathermic oil nanofluids for solar energy systems. Energy.

[28] Eapen J., Rusconi R., Piazza R., Yip S. (2010). The Classical Nature of Thermal Conduction in Nanofluids. J. Heat Transf..

[29] Afrand M. (2017). Experimental study on thermal conductivity of ethylene glycol containing hybrid nano-additives and development of a new correlation. Appl. Therm. Eng..

[30] Esfe M.H., Arani A.A.A., Rezaie M., Yan W.M., Karimipour A. (2015). Experimental determination of thermal conductivity and dynamic viscosity of Ag-MgO/water hybrid nanofluid. Int. Commun. Heat Mass Transf..

[31] Esfe M.H., Yan W.M., Akbari M., Karimipour A., Hassani M. (2015). Experimental study on thermal conductivity of DWCNT-ZnO/water-EG nanofluids. Int. Commun. Heat Mass Transf..

[32] Esfe M.H., Esfandeh S., Saedodin S., Rostamian H. (2017). Experimental evaluation, sensitivity analyzation and ANN modeling of thermal conductivity of ZnO-MWCNT/EG-water hybrid nanofluid for engineering applications. Appl. Therm. Eng..

[33] Esfe M.H., Arani A.A.A., Firouzi M. (2017). Empirical study and model development of thermal conductivity improvement and assessment of cost and sensitivity of EG-water based SWCNT-ZnO (30%:70%) hybrid nanofluid. J. Mol. Liq..

[34] Rostamian S.H., Biglari M., Saedodin S., Esfe M.H. (2017). An inspection of thermal conductivity of CuO-SWCNTs hybrid nanofluid versus temperature and concentration using experimental data, ANN modeling and new correlation. J. Mol. Liq..

[35] Das S.K., Choi S.U.S., Yu W., Pradeep T. (2007). Nanofluids: Science and Technology.

[36] Karthikeyan N.R., Philip J., Raj B. (2008). Effect of clustering on the thermal conductivity of nanofluids. Mater. Chem. Phys..

[37] Philip J., Shima P.D. (2012). Thermal properties of nanofluids. Adv. Colloid Interface Sci..

[38] Keblinski P., Phillpot S.R., Choi S.U.S., Eastman J.A. (2001). Mechanisms of heat flow in suspensions of nano-sized particles (nanofluids). Int. J. Heat Mass Transf..

[39] Loulijat H., Zerradi H. (2019). The effect of the liquid layer around the spherical and cylindrical nanoparticles in enhancing thermal conductivity of nanofluids. J. Heat Transf..

[40] Xue L., Keblinski P., Phillpot S.R., Choi S.U.S., Eastman J.A. (2004). Effect of liquid layering at the liquid-solid interface on thermal transport. Int. J. Heat Mass Transf..

[41] Das P.K. (2017). A review based on the effect and mechanism of thermal conductivity of normal nanofluids and hybrid nanofluids. J. Mol. Liq..

[42] Sharma S., Tiwari A.K., Tiwari S., Prakash R. (2018). Viscosity of hybrid nanofluids: Measurement and comparison. J. Mech. Eng. Sci..

[43] Ahammed N., Asirvatham L.G., Wongwises S. (2016). Effect of volume concentration and temperature on viscosity and surface tension of graphene-water nanofluid for heat transfer applications. J. Therm. Anal. Calorim..

[44] Murshed S.M.S., Estellé P. (2017). A state of the art review on viscosity of nanofluids. Renew. Sustain. Energy Rev..

[45] Hwang K.S., Lee J.H., Jang S.P. (2007). Buoyancy-driven heat transfer of water-based Al_2_O_3_ nanofluids in a rectangular cavity. Int. J. Heat Mass Transf..

[46] Bahrami M., Akbari M., Karimipour A., Afrand M. (2016). An experimental study on rheological behavior of hybrid nanofluids made of iron and copper oxide in a binary mixture of water and ethylene glycol: Non-Newtonian behavior. Exp. Therm. Fluid Sci..

[47] Nadooshan A.A., Eshgarf H., Afrand M. (2018). Measuring the viscosity of Fe_3_O_4_-MWCNTs/EG hybrid nanofluid for evaluation of thermal efficiency: Newtonian and non-Newtonian behavior. J. Mol. Liq..

[48] Motahari K., Moghaddam M.A., Moradian M. (2018). Experimental investigation and development of new correlation for influences of temperature and concentration on dynamic viscosity of MWCNT-SiO_2_ (20–80)/20W50 hybrid nano-lubricant. Chin. J. Chem. Eng..

[49] Zawawi N.N.M., Azmi W.H., Redhwan A.A.M., Sharif M.Z., Sharma K.V. (2017). Propriétés thermo-physiques du nanolubrifiant composite Al_2_O_3_-SiO_2_/PAG pour les systèmes frigorifiques. Int. J. Refrig..

